# Seed Priming with Carbon Nanomaterials Improves the Bioactive Compounds of Tomato Plants under Saline Stress

**DOI:** 10.3390/plants11151984

**Published:** 2022-07-30

**Authors:** Yolanda González-García, Elsy Rubisela López-Vargas, Marissa Pérez-Álvarez, Gregorio Cadenas-Pliego, Adalberto Benavides-Mendoza, Jesús Valdés-Reyna, Fabián Pérez-Labrada, Antonio Juárez-Maldonado

**Affiliations:** 1 Doctorado en Ciencias en Agricultura Protegida, Universidad Autónoma Agraria Antonio Narro, Saltillo 25315, Coahuila, Mexico; yolanda_glezg@hotmail.com (Y.G.-G.); lopez2690vargas@gmail.com (E.R.L.-V.); 2 Centro de Investigación en Química Aplicada, Saltillo 25294, Coahuila, Mexico; pamarissa@hotmail.com (M.P.-Á.); gregorio.cadenas@ciqa.edu.mx (G.C.-P.); 3 Departamento de Horticultura, Universidad Autónoma Agraria Antonio Narro, Saltillo 25315, Coahuila, Mexico; abenmen@gmail.com; 4 Departamento de Botánica, Universidad Autónoma Agraria Antonio Narro, Saltillo 25315, Coahuila, Mexico; jvaldes.reyna@gmail.com (J.V.-R.); fabperlab@outlook.com (F.P.-L.)

**Keywords:** nanotechnology, nanotubes, graphene, *Solanum lycopersicum* L., adverse environmental conditions

## Abstract

The consumption of food with a high content of bioactive compounds is correlated with the prevention of chronic degenerative diseases. Tomato is a food with exceptional nutraceutical value; however, saline stress severely affects the yield, the quality of fruits, and the agricultural productivity of this crop. Recent studies have shown that seed priming can mitigate or alleviate the negative effects caused by this type of stress. However, the use of carbon nanomaterials (CNMs) in this technique has not been tested for this purpose. In the present study, the effects of tomato seed priming with carbon nanotubes (CNTs) and graphene (GP) (50, 250, and 500 mg L^−1^) and two controls (not sonicated and sonicated) were evaluated based on the content of photosynthetic pigments in the leaves; the physicochemical parameters of the fruits; and the presence of enzymatic and non-enzymatic antioxidant compounds, carotenoids, and stress biomarkers such as hydrogen peroxide (H_2_O_2_) and malondialdehyde (MDA) in the leaves and fruits of tomato plants without saline stress and with saline stress (50 mM NaCl). The results show that saline stress in combination with CNTs and GP increased the content of chlorophylls (9.1–21.7%), ascorbic acid (19.5%), glutathione (≈13%), proteins (9.9–11.9%), and phenols (14.2%) on the leaves. The addition of CNTs and GP increased the activity of enzymes (CAT, APX, GPX, and PAL). Likewise, there was also a slight increase in the content of H_2_O_2_ (by 20.5%) and MDA (3.7%) in the leaves. Salinity affected the quality of tomato fruits. The physico-chemical parameters and bioactive compounds in both the stressed and non-stressed tomato plants were modified with the addition of CNTs and GP. Higher contents of total soluble solids (25.9%), phenols (up to 144.85%), flavonoids (up to 37.63%), ascorbic acid (≈28%), and lycopene (12.4–36.2%) were observed. The addition of carbon nanomaterials by seed priming in tomato plants subjected to saline stress modifies the content of bioactive compounds in tomato fruits and improves the antioxidant defense system, suggesting possible protection of the plant from the negative impacts of stress by salinity. However, analysis of the mechanism of action of CNMs through seed priming, in greater depth is suggested, perhaps with the use of omics sciences.

## 1. Introduction

Seed priming is a pre-sowing treatment in which the seeds are placed in a specific, defined solution concentration for a specified period [1]. Priming stimulates stress responses, through priming memory in the seeds, activating physiological and metabolic operations such as DNA repair pathways, de novo protein synthesis, reduction in metabolite leakage, and positive regulation of gene expression for the synthesis of antioxidant compounds [2]. These responses defend the cell against oxidative damage and lipid peroxidation, and allow plants to achieve a greater capacity to quickly and effectively combat different types of stress [3]. Shafiq et al. mentioned that the nano-priming of seeds (soaking of seeds in NMs) is a highly efficient and effective process due to the unique physicochemical properties that NMs possess [4]. Nano-priming triggers special metabolic processes, which are naturally activated and provide protection to seeds during storage, improving the germination, growth, production, and quality of crops, and increasing the resistance of crops to conditions of abiotic or biotic stress [5].

Various strategies have been sought to help plants mitigate the effects of salinity, among which the use of nanotechnology through the application of nanomaterials (NMs) to induce tolerance of salinity in plants stands out [6]. NMs can be applied in small amounts through different routes (foliar spray, seed, tubers, soil solution or nutrient solution in soilless systems, etc.) to induce greater tolerance of environmental stress and improve the nutraceutical quality of food [7]. NMs have the potential to be used as elicitors for the induction of bioactive compounds in plants by inducing the expression of genes involved in the biosynthesis of secondary metabolites [8]. They also have the ability to mitigate the limitations associated with abiotic and biotic stress by activating the defense system of plants through the formation of ROS and the accumulation of bioactive compounds, and triggering other key metabolic activities in plants stressed by salinity [9]. However, the effects of NMs on plants vary due to the plant species, the growth stage in which they are applied, the method, and the duration of exposure, among other things, and also depend on the intrinsic properties of the NMs, including the shape, size, chemical composition, concentration, surface structure, aggregation, and solubility [10]. NMs used include nanoparticles (NPs) of some metalloids and metal oxides (such as Ag, Cu, Si, Zn, B, Fe, and Mn), chitosan, and carbon nanomaterials (CNMs) such as carbon nanotubes (CNTs), graphene (GP), and fullerene. GP is “a two-dimensional crystal composed of mono layers of carbon atoms, arranged in a honeycomb-shaped network”. It was the first 2D material to become available [11]. CNTs are unique tubular nanostructures differing in their diameter, length, number of layers, and chirality. Both CNMs produce morphological, physiological, and biochemical responses that help the plant to tolerate stress, whether biotic or abiotic, by generating changes in the transcriptome, proteome, metabolome, and ionome [12]. The effects of CNMs on plants range from improved seed germination to increased productivity and crop yield [13]. At low concentrations, they activate the water channel (aquaporins), which improves water absorption, nutrient absorption, seed germination, seedling growth, and photosynthesis [14]. Conversely, in high doses, CNMs promote the production of free radicals, which induce oxidative stress and cell damage. Furthermore, CNMs stimulate the accumulation of bioactive compounds in a dose-dependent manner [13].

Tomato (*Solanum lycopersicum* L.) is one of the most important horticultural crops in the world due to its high nutritional and economic value [15]. It is an important source of bioactive compounds such as carotenoids (lycopene and β-carotene), phenolic compounds, vitamins like vitamin C, vitamin A, and vitamin B [16]. The consumption of foods with a high content of bioactive compounds is correlated with the prevention of human chronic degenerative diseases, cardiovascular diseases, cancer, and neurodegenerative diseases by reducing the oxidative damage of important biomolecules such as membrane lipids, enzymatic proteins, and DNA [17].

Currently, the main challenges facing global agriculture include climate change, urbanization, environmental problems such as drought, salinity of water and soils, and the accumulation of pesticides and fertilizers [5]. These problems are further intensified by an alarming increase in the demand for food necessary to feed an estimated world population of 9.7 billion by 2050 and 10.9 billion by 2100 [15]. For this reason, an increase of between 60 and 110% in world food production is required [18]. A severe loss in agricultural yield and productivity has been recorded as a result of saline stress, which affects germination, vegetative growth, fruit formation, development, maturation, and quality [19,20]. Saline stress can cause ionic stress, osmotic stress, and oxidative stress in plants, and it also alters hormonal homeostasis and causes an imbalance in nutrients [9]. Under conditions of saline stress, it has been shown that there is an increase in reactive oxygen species (ROS) [21]. A high content of these ROS causes oxidative degradation of biomolecules such as lipids and proteins, DNA damage, and, as a result of lipid peroxidation breaking the cell membrane, greater permeability and ion leakage, and an increase in the malondialdehyde (MDA) content [18].

Plants have several mechanisms to cope with saline stress, which include ROS homeostasis, an increase in the antioxidant defense system, activation of ROS elimination pathways, compartmentalization of toxic ions, osmolyte biosynthesis, and ion homeostasis [22]. To counteract oxidative stress caused by ROS, plants have enzymatic and non-enzymatic detoxification systems, which are more active when plants are under stress [18].

In this context, the priming of tomato seeds with different concentrations of CNMs was carried out in order to evaluate the biochemical responses of tomato plants subjected to saline stress, emphasizing the antioxidant system and bioactive compounds. It may be possible that CNMs applied trough seed priming at an optimal concentration can act as elicitors and activate the antioxidant defense system to counteract the negative effects caused by saline stress while increasing the production of bioactive compounds. Although this technique has been used previously, the use of CNMs has not been evaluated for the purposes presented in this study.

## 2. Results

### 2.1. Content of Photosynthetic Pigments in Leaves

**Figure 1:**
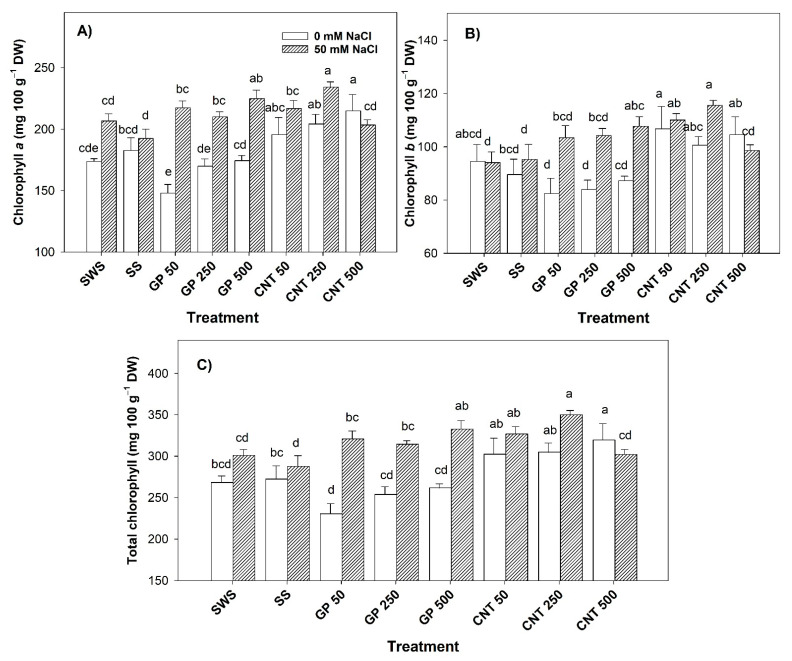
Photosynthetic pigments of tomato leaves treated with carbon nanomaterials and salinity: (**A**) chlorophyll *a*, (**B**): chlorophyll *b*, and (**C**) total chlorophyll. DW: dry weight; SWS: non-sonicated seeds; SS: sonicated seeds; GP: graphene; CNT: carbon nanotubes; 50, 250, and 500 represent the mg L^−1^ applied of carbon nanomaterial. Different letters indicate a significant difference between treatments according to Fisher (α = 0.05). *n* = 6 ± standard error.

Without salt stress, the CNT 250 and CNT 500 treatments increased chlorophyll *a* by 17.58% and 23.78%, respectively, compared to the non-sonicated control. Compared to the sonicated control, the CNT 500 treatment induced an increase of 17.63% (Figure 1A). Chlorophyll *b* increased by 19.17% with the CNT 50 treatment only compared with the sonicated control (Figure 1B). Total chlorophylls increased by 19.16% with the CNT 500 treatment with respect to the non-sonicated control, and 17.32% with respect to the sonicated control (Figure 1C).

Under saline stress, the GP 500 and CNT 250 treatments increased the chlorophyll content by 8.75% and 13.33% with respect to the non-sonicated control. Compared to the sonicated control, it was observed that the GP in its different doses presented an increase of 9.16–16.81%, and the CNT (50 and 250 mg L^−1^) an increase of 12.61–21.73%, respectively (Figure 1A). The GP 500, CNT 50, and CNT 250 treatments increased chlorophyll *b* by 14.57%, 17.01%, and 22.89%, respectively, compared to the non-sonicated control, and by 13.11%, 15.52%, and 21.33%, respectively, compared to the sonicated control (Figure 1B). Total chlorophylls increased by 10.57%, 8.65%, and 16.32% with GP 500, CNT 50, and CNT 250, respectively, with respect to the non-sonicated control (Figure 1C).

### 2.2. Stress Biomarkers in Tomato Leaves

**Figure 2:**
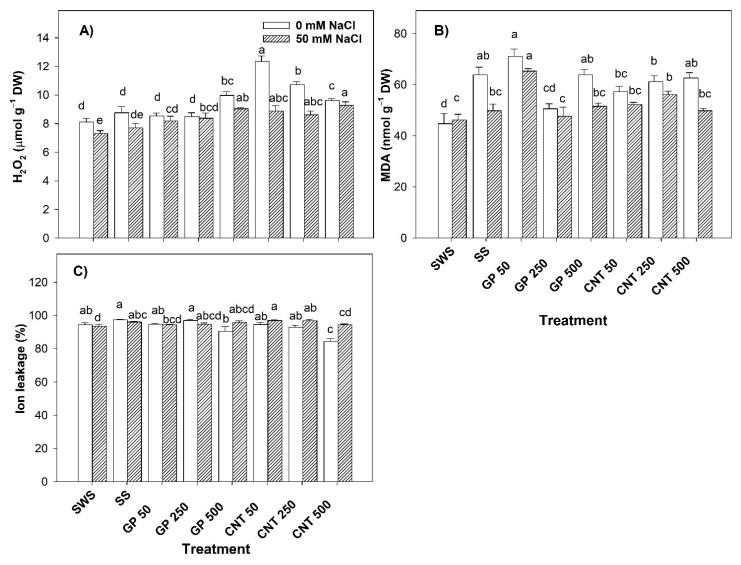
Stress markers in tomato plant leaves treated with carbon nanomaterials and salinity: (**A**) hydrogen peroxide (H_2_O_2_), (**B**) malondialdehyde (MDA), and (**C**) ion leakage. DW: dry weight; SWS: non-sonicated seeds; SS: sonicated seeds; GP: graphene; CNT: carbon nanotubes; 50, 250, and 500 represent the mg L^−1^ applied of carbon nanomaterial. Different letters indicate a significant difference between treatments according to Fisher (α = 0.05). *n* = 6 ± standard error.

The results regarding the hydrogen peroxide (H_2_O_2_) and malondialdehyde (MDA) content and the ion leakage (%) in tomato leaves revealed significant differences between treatments (Figure 2). Without saline stress, H_2_O_2_ increased with the addition of CNTs in different doses (50, 250, and 500 mg L^−1^), with CNT 50 being the one resulting in the greatest increase of 52.40% compared to the non-sonicated control and 41.10% compared to the sonicated control. The GP with the dose of 500 mg L^−1^ increased by 22.93% with respect to the non-sonicated control, and 13.81% with respect to the sonicated control (Figure 2A). The MDA increased with the GP 50 application by 58.91% compared to the non-sonicated control. Regarding the sonicated control, the GP 250 treatment only decreased the MDA content by 20.82% (Figure 2B). The ion leakage decreased by 10.71% with the addition of CNT 500 compared to the non-sonicated control, and compared to the sonicated control, it decreased by 13.50%. The GP 500 treatment also decreased the MDA content by 7.10% with respect to the non-sonicated control (Figure 2C).

Under saline stress, with the GP 500 treatment, H_2_O_2_ increased by 23.50% and with CNT 500 by 26.78% with respect to the non-sonicated control, and by 17.40% with GP 500 and by 20.52% with CNT 500 with respect to the sonicated control (Figure 2A). The addition of GP 50 resulted in an increase of 41.38% with respect to the non-sonicated control and 31.08% with respect to the sonicated control while the addition of CNT 250 mg L^−1^ resulted in an increase of 21.47% with respect to the non-sonicated control (Figure 2B). MDA increased by 3.77% when adding CNT 50 and 3.65% with CNT 250. In addition, the sonicated control induced greater ion loss (2.75%) compared to the non-sonicated control (Figure 2C).

### 2.3. Proteins and Enzymatic Activity in Leaves

**Figure 3:**
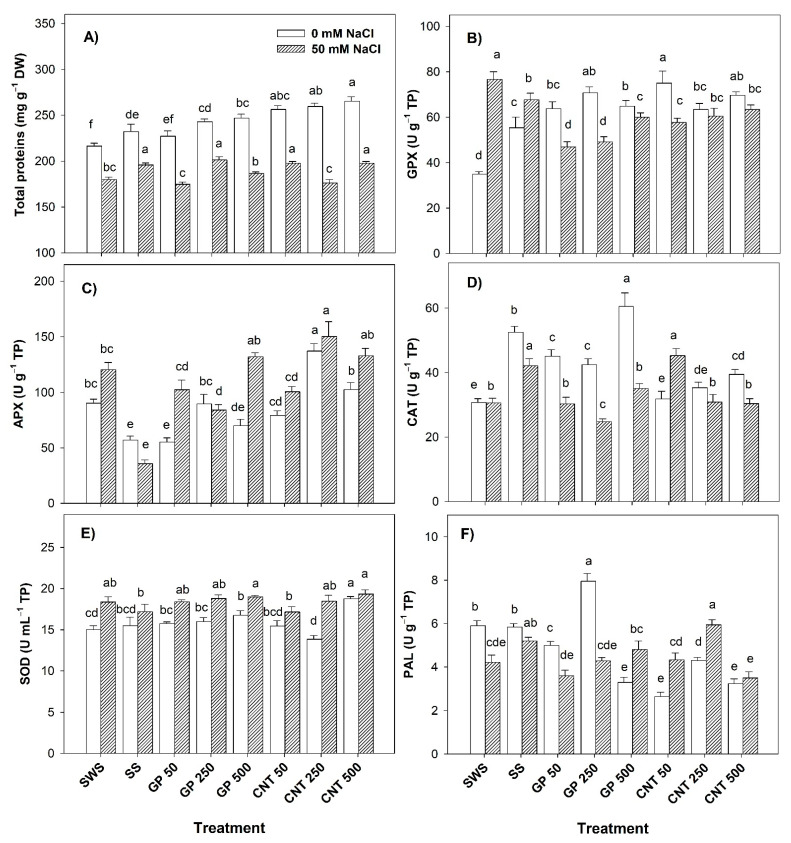
Total proteins and enzymatic activity in tomato leaves treated with carbon nanomaterials and salinity: (**A**) total proteins, (**B**) glutathione peroxidase (GPX), (**C**) ascorbate peroxidase (APX), (**D**) catalase (CAT), (**E**) superoxide dismutase (SOD), and (**F**) phenylalanine ammonia lyase (PAL). DW: dry weight; SWS: non-sonicated seeds; SS: sonicated seeds; GP: graphene; CNT: carbon nanotubes; 50, 250, and 500 represent the mg L^−1^ applied of carbon nanomaterial. Different letters indicate a significant difference between treatments according to Fisher (α = 0.05). *n* = 6 ± standard error.

Without salt stress, the protein content increased with the CNT 500 treatment by 22.57% and 14.25% compared to the non-sonicated control and the sonicated control, respectively. In addition, the sonicated control increased the protein content by 7.28% compared to the non-sonicated control (Figure 3A). The activity of GPX increased with the different doses of GP and CNTs, with CNT 50 presenting the highest value of 114.47% and 35.56% with respect to the non-sonicated control and sonicated control, respectively (Figure 3B). The addition of CNT 250 increased the APX activity by 51.87% compared to the non-sonicated control. Meanwhile, when comparing the treatments with the sonicated control, the APX activity increased by 140.26% with GP 250 and by 56.98% with CNT 250 (Figure 3C). CAT activity increased with the addition of GP at all doses in a range of 46–97% while CNT 500 increased it by 28.43% with respect to the non-sonicated control. Compared to the sonicated control, GP 500 only increased CAT activity by 15.39% (Figure 3D). With GP 500, SOD activity increased by 11.60% and by 25.00% with CNT 500 with respect to the non-sonicated control (Figure 3E). The GP 250 treatment increased PAL activity by 34.75% and 36.13% compared to the non-sonicated control and the sonicated control, respectively (Figure 3F).

Under saline conditions, the proteins increased by 11.93% with GP 250 and 9.91% with CNT 50 and CNT 500 with respect to the non-sonicated control. With respect to the sonicated control, the protein content decreased with the addition of GP 50 (10.78%), GP 500 (4.77%), and CNT 250 (10.00%) (Figure 3A). The application of the treatments with GP and CNTs decreased the activity of GPX with respect to both controls (Figure 3B). The APX enzyme showed higher activity with CNT 250, with an increase of 24.73% compared to the non-sonicated control. With respect to the sonicated control, all concentrations of GP and CNTs increased the activity of APX (Figure 3C). The CNT 50 treatment increased CAT activity by 47.83% compared to the non-sonicated control. Meanwhile, with respect to the sonicated control, the CAT activity decreased significantly with all treatments (Figure 3D). The GP 500 and CNT 500 treatments increased SOD activity by 10.54% and 12.58%, respectively, compared to the sonicated control (Figure 3E). PAL activity increased by 41.09% only with CNT 250 with respect to the non-sonicated control (Figure 3F).

### 2.4. Non-Enzymatic Antioxidant Compounds in Leaves

**Figure 4:**
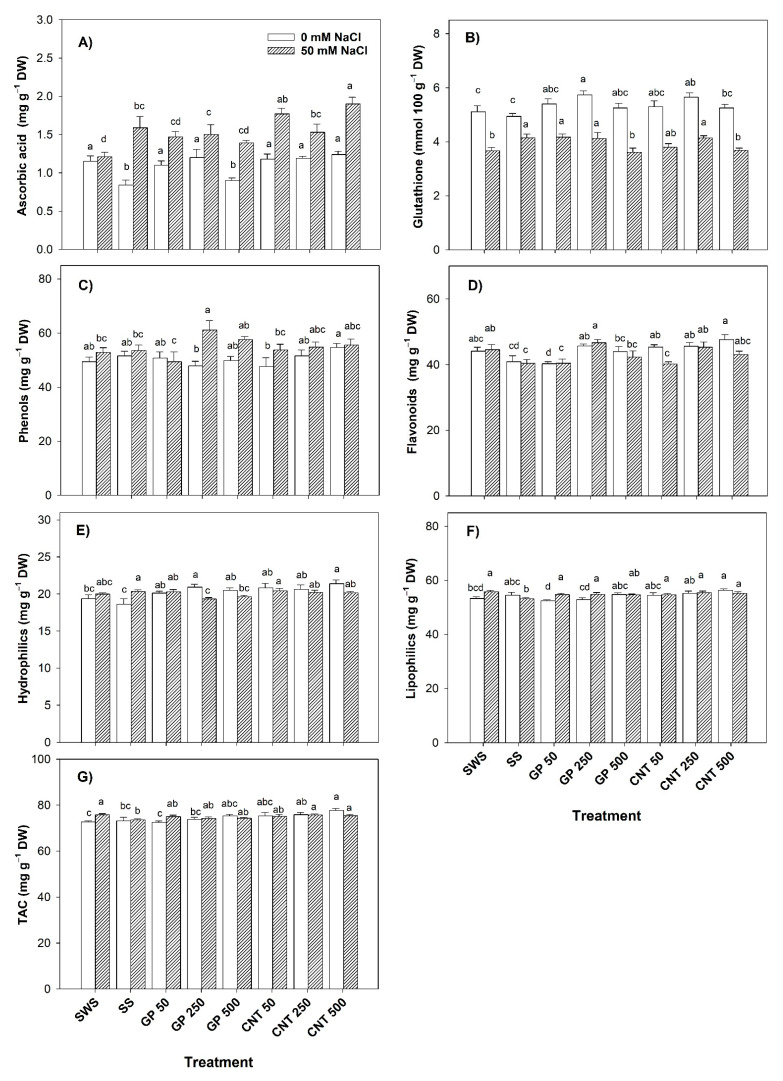
Non-enzymatic antioxidant compounds in tomato plant leaves treated with carbon nanomaterials and salinity: (**A**) ascorbic acid, (**B**) glutathione, (**C**) phenols, (**D**) flavonoids, (**E**) hydrophilics antioxidant capacity, (**F**) lipophilics antioxidant capacity, and (**G**) total antioxidant capacity (TAC). DW: dry weight; SWS: non-sonicated seeds; SS: sonicated seeds; GP: graphene; CNT: carbon nanotubes; 50, 250, and 500 represent the mg L^−1^ applied of carbon nanomaterial. Different letters indicate a significant difference between treatments according to Fisher (α = 0.05). *n* = 6 ± standard error.

Without salt stress, ascorbic acid decreased with the addition of GP 500 (21.74%) compared to the non-sonicated control. However, with respect to the sonicated control, all treatments increased the content of ascorbic acid in the range of 30.95–47.62%, except for GP 500, which did not show any differences (Figure 4A). GSH increased by 12.13% and 10.57% with GP 250 and CNT 250 with respect to the non-sonicated control while with respect to the sonicated control, it increased by 15.99% and 14.37%, respectively (Figure 4B). Phenols did not show differences between treatments (Figure 4C). Flavonoids decreased by 8.58% with the GP 50 treatment compared to the non-sonicated control while compared to the sonicated control, they increased by 11.56% with GP 250, and by up to 16.28% with all CNT doses (Figure 4D). The antioxidant capacity of the hydrophilic compounds increased by 8.00% with GP 250 and by 10.43% with CNT 500 with respect to the non-sonicated control. Compared with the sonicated control, all treatments induced an increase, with GP 250 and CNT 500 showing the greatest increase (12.47% and 15.00%, respectively) (Figure 4E). The antioxidant capacity of lipophilic compounds increased by 5.63% with CNT 500 with respect to the non-sonicated control but decreased by 3.91% with GP 50 with respect to the sonicated control (Figure 4F). The total antioxidant capacity increased with CNT 250 and CNT 500 by 4.36% and 6.90%, respectively, compared to the non-sonicated control, and CNT 500 increased by 6.20% with respect to the sonicated control (Figure 4G).

Under saline stress, ascorbic acid increased by 23.97% with the addition of GP 250 and by 26–57% with all CNT doses with respect to the non-sonicated control. Meanwhile, the CNT 500 treatment only increased the ascorbic acid content by 19.50% with respect to the sonicated control (Figure 4A). GSH increased with GP 50 (13.62%) and GP 250 (12.53%) with respect to the non-sonicated control and decreased with GP 500 (13.01%) with respect to the sonicated control. Meanwhile, with CNT 250, an increase of 12.80% was observed with respect to the non-sonicated control, and with respect to the sonicated control, it decreased with CNT 500 (11.33%) (Figure 4B). Phenols were increased by 15.80% with the GP 250 treatment with respect to the non-sonicated control and by 14.22% with respect to the sonicated control (Figure 4C). Flavonoids decreased compared to the non-sonicated control with GP 50 (9.14%) and with CNT 50 (9.77%). Compared to the sonicated control, the flavonoids content was increased with GP 250 (15.42%) and CNT 250 (11.98%) (Figure 4D). The addition of GP (250 and 500 mg L^−1^) decreased the antioxidant capacity of the hydrophilic compounds (4.92% and 3.49%, respectively) compared to the sonicated control (Figure 4E). With the exception of GP 500, all treatments induced an increase in the antioxidant capacity of the lipophilic compounds compared to the sonicated control in the range of 2.78–4.23% (Figure 4F). The CNT 250 and CNT 500 treatments increased the total antioxidant capacity by 2.92% and 2.47%, respectively, compared to the sonicated control (Figure 4G).

### 2.5. Physico-Chemical Characteristics of the Fruits

**Figure 5:**
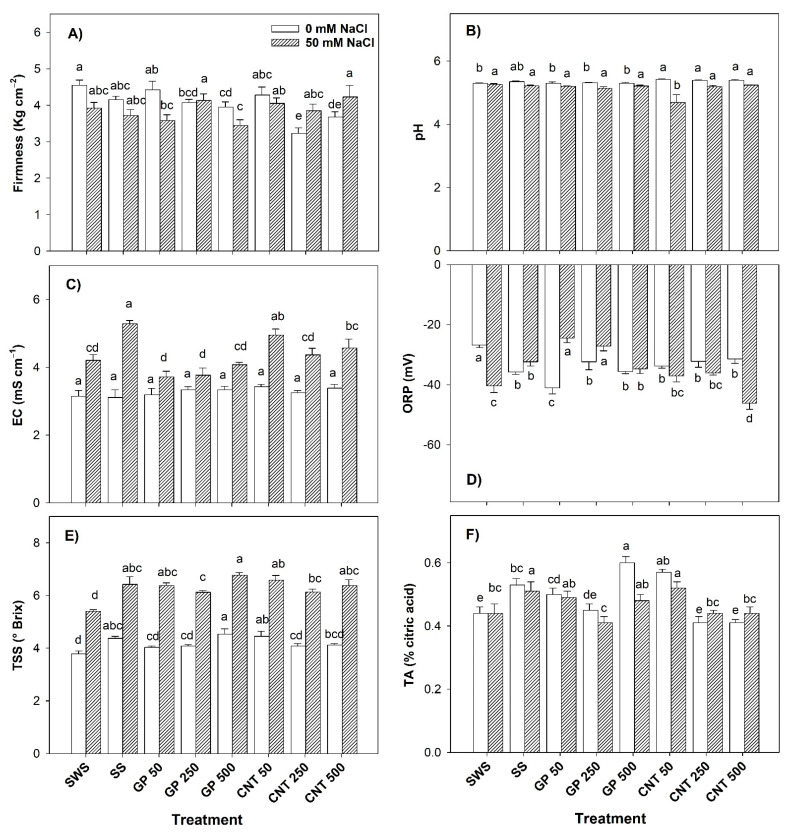
Physico-chemical characteristics of the fruits of tomato plants treated with carbon nanomaterials and salinity: (**A**) firmness, (**B**) hydrogen potential (pH), (**C**) electrical conductivity (EC), (**D**) oxidation-reduction potential (ORP), (**E**) total soluble solids (TSS), and (**F**) titratable acidity (TA). SWS: non-sonicated seeds; SS: sonicated seeds; GP: graphene; CNT: carbon nanotubes; 50, 250, and 500 represent the mg L^−1^ applied of carbon nanomaterial. Different letters indicate a significant difference between treatments according to Fisher (α = 0.05). *n* = 6 ± standard error.

Without salinity, the firmness of the fruits decreased with the addition of 250 and 500 mg L^−1^ of GP (10.87% and 13.04%) and CNTs (30.43% and 19.57%) compared to the non-sonicated control. Compared to the sonicated control, the doses of 250 and 500 mg L^−1^ of CNTs decreased the firmness of the fruits by 23.81% and 11.90%, respectively (Figure 5A). The hydrogen potential (pH) of the fruits increased with all CNTs doses by up to 2.26% with respect to the non-sonicated control (Figure 5B). There were no differences between treatments in terms of the electrical conductivity of the fruits (Figure 5C). The oxidation-reduction potential (ORP) in the fruits decreased with all treatments. GP 50 presented the greatest decrease of 52.81% with respect to the non-sonicated control and 14.94% with respect to the sonicated control (Figure 5D). The total soluble solids (TSS) of the fruits increased with the GP 500 (18.42%) and CNT 50 (18.42%) treatments with respect to the non-sonicated control (Figure 5E). The titratable acidity (TA) of the fruits increased by 13.64% and 36.36% with GP 50 and GP 500, respectively, and by 29.55% with CNT 50 with respect to the non-sonicated control. Compared to the sonicated control, GP 500 increased TA by 13.21% (Figure 5F).

Under saline stress, the firmness of the fruits was not affected by the CNMs (Figure 5A). The pH of fruits decreased with CNT 50 by 11.00% and 10.15% compared to the non-sonicated control and the sonicated control, respectively (Figure 5B). EC increased by 17.58% with CNT 50 with respect to the non-sonicated control. Compared to the sonicated control, all treatments decreased the electrical conductivity (EC) of the fruits, except CNT 50 (Figure 5C). The ORP of the fruits increased with all the doses of GP in a range of 14.03–39.25% with respect to the non-sonicated control. Compared to the sonicated control, only the 50 and 250 mg L^−1^ doses of GP increased the ORP by 24.22% and 15.96%, respectively, while CNT 500 decreased it by 42.81% (Figure 5D). The TSS of the fruits increased with all treatments, with GP 500 resulting in the highest increase of 25.93% compared to the non-sonicated control (Figure 5E). The TA of the fruits increased by 18.18% with CNT 50 with respect to the non-sonicated control. Compared to the sonicated control, TA decreased by 19.61% with GP 250 and by 13.73% with CNT 250 and CNT 500 (Figure 5F).

When comparing only the non-sonicated control against the sonicated control in a condition without salinity, a significant difference was observed: the ORP decreased by 32.95% and the TSS sand the TA increased by 15.79% and 20.45%, respectively. In addition, under saline conditions, increases were observed in ORP (19.84%), EC (25.42%), TSS (18.52%), and TA (15.91%) in the non-sonicated control (Figure 5). This indicates that the mere act of carrying out the sonication process induces responses in the physico-chemical quality of the fruits.

### 2.6. Bioactive Compounds in Fruits

**Figure 6:**
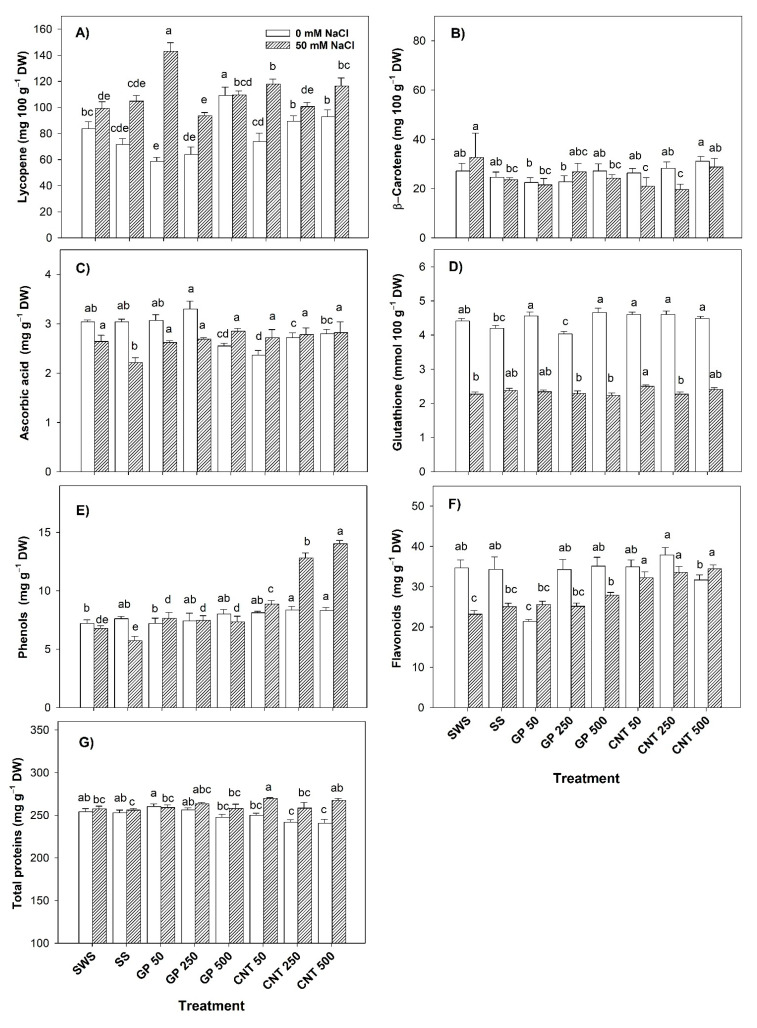
Bioactive compounds in fruits of tomato plants treated with carbon nanomaterials and salinity: (**A**) lycopene, (**B**) β-carotene, (**C**) ascorbic acid, (**D**) glutathione, (**E**) phenols, (**F**) flavonoids, (**G**) total proteins. DW: dry weight; SWS: non-sonicated seeds; SS: sonicated seeds; GP: graphene; CNT: carbon nanotubes; 50, 250, and 500 represent the mg L^−1^ applied of carbon nanomaterial. Different letters indicate a significant difference between treatments according to Fisher (α = 0.05). *n* = 6 ± standard error.

Without salt stress, the addition of GP 500 increased the lycopene content by 30.49% with respect to the non-sonicated control. Compared to the sonicated control, the GP 500 increased the lycopene content by 52.33% while the CNT 250 and CNT 500 treatments increased it by 24.68% and 29.55%, respectively (Figure 6A). β-carotene increased by 10.31% and 26.35% with the GP 500 and CNT 500 treatments with respect to the sonicated control (Figure 6B). The GP 500, CNT 50, and CNT 250 treatments decreased the content of ascorbic acid while the rest of the treatments had no effect (Figure 6C). GSH increased with all GP and CNT treatments except GP 250 compared to the sonicated control (Figure 6D). Phenols increased with CNT 250 (16.11%) and CNT 500 (15.41%) compared to the non-sonicated control (Figure 6E). Flavonoids only decreased with GP 50 with respect to both controls while in the rest of the treatments, there were no differences (Figure 6F). Proteins decreased with CNT 250 and CNT 500 in comparison to both controls while the rest of the treatments were the same as the controls (Figure 6G).

Under saline conditions, lycopene increased with the GP 50, CNT 50, and CNT 500 treatments by 43.99%, 18.88%, and 17.38%, respectively, compared to the non-sonicated control. Compared to the sonicated control, the GP 50 and CNT 50 treatments increased the lycopene content by 36.25% and 12.49%, respectively (Figure 6A). The β-carotene content was reduced by 39.75% with CNT 250 compared to the non-sonicated control. In comparison to the sonicated control, there were no differences between treatments (Figure 6B). Ascorbic acid was increased with GP 500 and CNT 500 by 28.96% and 27.60%, respectively, compared to the sonicated control (Figure 6C). GSH increased by 10.13% with CNT 50 compared to the non-sonicated control (Figure 6D). Phenols increased by up to 107.24% with CNT 500 with respect to the non-sonicated control. Compared to the sonicated control, the increase in the phenols content was 144.85% with CNT 500 (Figure 6E). The GP 500 induced a higher content of flavonoids compared to the non-sonicated control (20.48% more); however, the effect was greater with CNTs, since all doses increased this variable by 38.94–48.56%. Compared to the sonicated control, only the CNTs increased the flavonoids content up to 37.63% (Figure 6F). The CNT 50 induced 4.65% more proteins compared to the non-sonicated control. Compared to the sonicated control, CNT 50 and CNT 500 increased proteins by 5.29% and 4.45%, respectively (Figure 6G).

### 2.7. Antioxidant Capacity in Fruits

**Figure 7:**
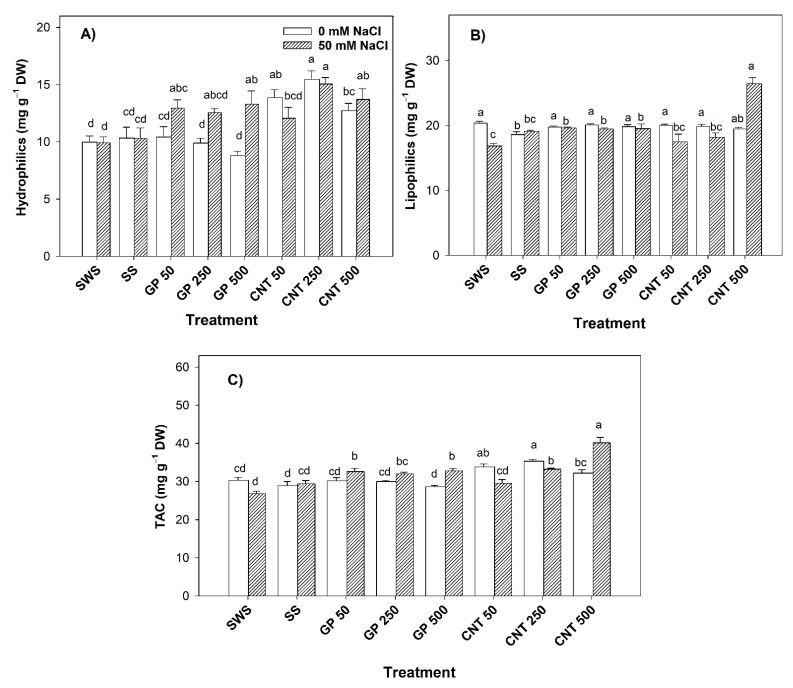
Antioxidant capacity in fruits of tomato plants treated with carbon nanomaterials and salinity: (**A**) hydrophilics antioxidant capacity, (**B**) lipophilics antioxidant capacity, and (**C**) total antioxidant capacity (TAC). DW: dry weight; SWS: non-sonicated seeds; SS: sonicated seeds; GP: graphene; CNT: carbon nanotubes; 50, 250, and 500 represent the mg L^−1^ applied of carbon nanomaterial. Different letters indicate a significant difference between treatments according to Fisher (α = 0.05). *n* = 6 ± standard error.

Without salinity, the antioxidant capacity of hydrophilic compounds increased by 27.56–55.01% with CNTs compared to the non-sonicated control. With respect to the sonicated control, it increased by 34.04% with CNT 50 and by 49.61% with CNT 250 (Figure 7A). The antioxidant capacity of lipophilic compounds increased by up to 7.96% with all GP and CNTs treatments except CNT 500 with respect to the sonicated control (Figure 7B). The total antioxidant capacity of the GP treatments was unaffected. Meanwhile, the CNT 50 and CNT 500 treatments increased the total antioxidant capacity by 11.57% and 16.45% compared to the non-sonicated control. Compared to the sonicated control, all doses of CNTs increased this variable by up to 22.12% with CNT 250 (Figure 7C).

Under salinity, the antioxidant capacity of hydrophilic compounds increased with GP 50 (30.54%), GP 500 (33.97%), CNT 250 (51.81%), and CNT 500 (38.21%) compared to the non-sonicated control. Compared to the sonicated control, it was increased with GP 500 (28.78%), CNT 250 (45.92%), and CNT 500 (32.85%) (Figure 7A). The different doses of GP increased the antioxidant capacity of lipophilic compounds (15.49–16.50%); however, CNT 500 presented the greatest increase of 56.91% compared to the non-sonicated control. Compared to the sonicated control, CNT 500 presented an increase of 38.43% (Figure 7B). Both CNMs presented positive effects. The different doses of GP increased the total antioxidant capacity by up to 22.49% with respect to the non-sonicated control and by 11.45% with respect to the sonicated control. The CNTs increased the total antioxidant capacity by up to 49.94% with respect to the non-sonicated control and by 36.44% with respect to the sonicated control (Figure 7C).

**Figure 8:**
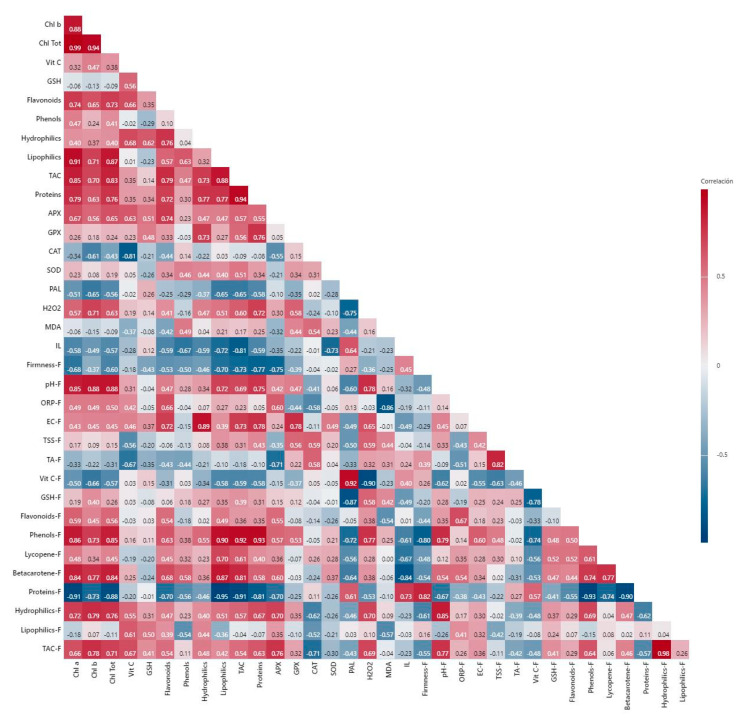
Pearson’s correlation of all variables obtained from non-stressed plants. Chl a: chlorophyll a; Chl b: chlorophyll b; Chl Tot: total chlorophylls; Vit C: vitamin C; GSH: glutathione; Hydrophilics: hydrophilic antioxidant capacity; Lipophilics: lipophilic antioxidant capacity; TAC: total antioxidant capacity; APX: ascorbate peroxidase; GPX: glutathione peroxidase; CAT: catalase; SOD: superoxide dismutase; PAL: phenylalanine ammonia lyase; MDA: malondialdehyde; IL: ion leakage; ORP: oxidation reduction potential; EC: electrical conductivity; TSS: total soluble solids; TA: titratable acidity. The “F” in front of each word represents each variable determined in fruits.

Without salinity, positive correlations were observed between chlorophylls and the lipophilic antioxidant capacity, total antioxidant capacity, flavonoids, and proteins in the leaves, all with indices ≥73. Furthermore, flavonoids presented a positive correlation with the hydrophilic antioxidant capacity (0.76), total antioxidant capacity (0.79), proteins (0.72), and APX activity (0.74). Meanwhile, the proteins correlated positively with the total antioxidant capacity (0.94). The most relevant negative correlations were observed between CAT activity and vitamin C (−0.81) and between ion leakage and total antioxidant capacity (−0.81). In the fruits, there was a positive correlation between firmness and proteins (0.82), and between pH and phenols (0.79) and total antioxidant capacity (0.85). In addition, a positive correlation between β-carotene and phenols was observed (0.74). In contrast, a negative correlation was observed between proteins and phenols (−0.93) and β-carotene (−0.90). A negative correlation was also observed between firmness and phenols (−0.80), and vitamin C and GSH (−0.78) (Figure 8).

**Figure 9:**
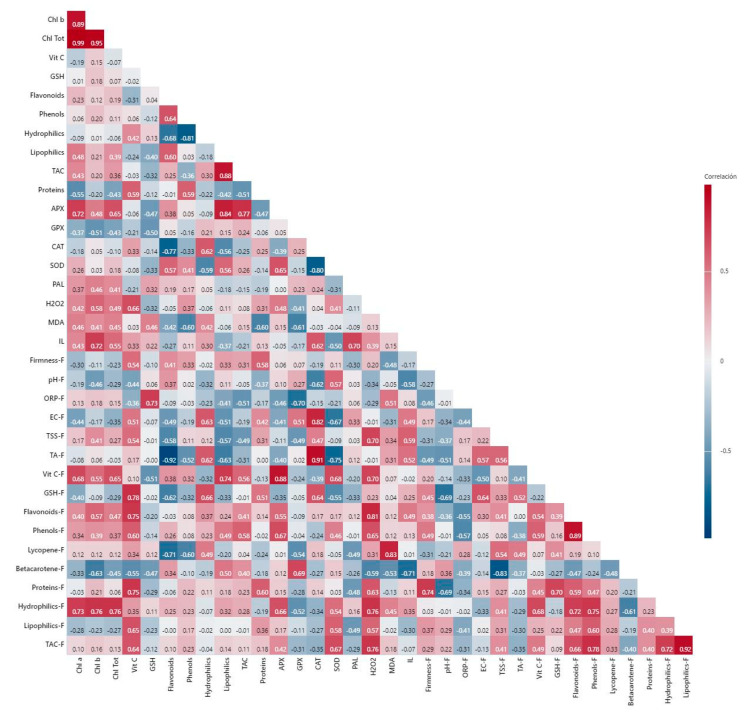
Pearson’s correlation of all variables obtained from stressed plants. Chl a: chlorophyll a; Chl b: chlorophyll b; Chl Tot: total chlorophylls; Vit C: vitamin C; GSH: glutathione; Hydrophilics: hydrophilic antioxidant capacity; Lipophilics: lipophilic antioxidant capacity; TAC: total antioxidant capacity; APX: ascorbate peroxidase; GPX: glutathione peroxidase; CAT: catalase; SOD: superoxide dismutase; PAL: phenylalanine ammonia lyase; MDA: malondialdehyde; IL: ion leakage; ORP: oxidation reduction potential; EC: electrical conductivity; TSS: total soluble solids; TA: titratable acidity. The “F” in front of each word represents each variable determined in fruits.

Under saline conditions, the most relevant positive correlations in leaves were observed between chlorophyll *b* and ion leakage (0.72) and between chlorophyll *a* and APX activity (0.72). In contrast, a negative correlation was observed between CAT activity and flavonoids (−0.77), and between phenols and hydrophilic antioxidant capacity (−0.81). Furthermore, SOD activity was negatively correlated with CAT activity (−0.80). In the fruits, firmness was positively correlated with proteins (0.74), flavonoids were positively correlated with phenols and hydrophilic antioxidant capacity (0.89 and 0.72, respectively), and phenols with hydrophilic antioxidant capacity and total antioxidant capacity (0.75 and 0.78, respectively). In contrast, a negative correlation was observed between total soluble solids and β-carotene (−0.83) (Figure 9).

## 3. Discussion

Saline stress causes a negative impact on several biochemical and physiological processes due to the increase in the production of ROS or reactive nitrogen species (RNS). Soltabayeva et al. mentioned that during saline stress, there is an increase in the enzymatic activity of CAT, SOD, APX, and GPX, and in bioactive compounds such as ascorbic acid and GSH, among others [21]. In addition, increases in stress biomarkers such as H_2_O_2_, MDA, and ion leakage are also observed during saline stress. H_2_O_2_ is a signaling molecule associated with the initiation of stress in plants. Under conditions of saline stress, H_2_O_2_ acts as a signaling agent, causing an ionic balance in plant cells and consequently resistance to it [23]. However, a high content of H_2_O_2_ causes oxidative degradation of biomolecules due to lipid peroxidation, which breaks the cell membrane, causing greater permeability, greater ion leakage, and an increase in the content of MDA [18]. An increased MDA content is a saline stress response in various crops and is a sign of membrane damage at the cellular level under saline stress [2].

On the other hand, the application of CNMs through seed priming is an important, simple, profitable, and safe practice due to its low environmental impact and the positive impacts it can have on agriculture. Uptake and internalization of CNMs into plant cells through this technique has been demonstrated, resulting in numerous interactions between plants and CNMs, which can cause subtle to marked changes in biochemical, physiological, and biological properties and plant genetics [24,25]. CNMs can penetrate the seed coats by forming new pores that subsequently allow the entry of water, oxygen, nutrients, and external molecules into the interior of the seed, facilitating the germination process [13,26]. Moreover, it can increase the rate of survival, growth, development, and biomass production and induce a positive effect on the plant response to stress [13]. In addition, CNMs can also induce the formation of water channels (aquaporins) in seed coats by upregulating genes encoding aquaporin proteins [27,28] and activating the expression of genes that regulate cell division and cell wall extension [26]. This is ultimately reflected in a higher rate of growth and productivity of plants and a greater ability to tolerate stress.

One of the mechanisms of adaptation to stress is the production of antioxidant compounds (which can be bioactive compounds) and increased activity of the enzymes related to the inactivation of ROS [29]. Antioxidant compounds are biomolecules that prevent the oxidation of other molecules by inhibiting the initiation and elongation of the oxidative chain reaction of ROS [6]. These compounds are considered the first line of cellular defense used to prevent damage by ROS such as singlet oxygen (^1^O_2_), superoxide anion (O_2_^•–^), H_2_O_2_, or hydroxyl radicals (OH^•–^) [30]. The antioxidant defense system of plants includes non-enzymatic compounds, such as ascorbic acid (vitamin C), glutathione, alkaloids, carotenoids (lycopene and β-carotene), flavonoids, phenolic compounds, and tocopherols [31]. Meanwhile, the enzymatic compounds include enzymes such as SOD, CAT, APX, GPX, mono dehydroascorbate reductase (MDHAR), dehydroascorbate reductase (DHAR), glutathione reductase (GR), glutathione transferase (GST), guaiacol peroxidases (POX), and proteins similar to nicotinamide adenine dinucleotide phosphate (NADPH), among others [32]. This defense machinery of plants is more active when subjected to stress [18]. Under conditions of saline stress, it has been shown that there is an increase in ROS [21]. The SOD enzyme is the initial line of defense, as it catalyzes the dismutation of the superoxide radical (O_2_^•–^) to hydrogen peroxide (H_2_O_2_) and molecular oxygen (O_2_). The enzymes APX, GPX, and CAT participate in the transformation of H_2_O_2_ to H_2_O and oxygen. APX requires ascorbic acid and reduced glutathione as substrates while GPX uses GSH as a reducing agent. CAT is highly specific for H_2_O_2_ and does not require an activity reducer. An increase in the enzymatic activity of CAT effectively eliminates ROS produced during salt stress (mainly in the peroxisome) in plants [19]. Meanwhile, the PAL enzyme catalyzes L-phenylalanine to transcinamic acid to produce secondary metabolites, such as lignin, flavonoids, and other compounds, products of the phenylpropanoid pathway, which are also antioxidants [33]. 

The NMs applied in seeds enter through the parenchymal intercellular spaces of the cover until they reach the cotyledon. Once inside, the absorption of NMs in the cotyledons is regulated by aquaporins [34]. However, the hard coating of the seeds prevents the penetration of the NMs into the interior of the seed. As an alternative, some authors have proposed the sonication of seeds, since it has been shown that this process improves the fluidity of the cell wall by creating micropores or microcracks, which facilitate the entry of both water and nutrients into the endosperm of the seed [35]. In addition, it has been reported that by sonicating the seeds together with the CNMs, not only is the entry of the CNMs facilitated but the dispersion of the nanomaterial is also improved, which avoids agglomerations [36].

It has been reported that through imbibition, some NMs, such as CNTs, can break down the hard layer of the seed and create pores to enter the seed [36]. The seed priming with NMs activates a series of physiological and metabolic responses, such as an increase in gene expression to synthesize bioactive compounds, and enzymatic antioxidant compounds that regulate ROS homeostasis [2]. These responses increase the antioxidant system of plants, which allows a greater capacity to mitigate the negative effects of any stress and induce tolerance [3]. In general, nano-priming of seeds increases the resistance of crops to abiotic or biotic stress conditions [5]. CNMs can be internalized into chloroplasts, modify their shape, and increase their size, positively influencing the production of photosynthetic pigments such as chlorophylls and accessory pigments such as carotenoids and lycopene [33]. In seed priming, this is of utmost importance because chloroplasts are not only organelles for autotrophic growth but are also necessary during the seed germination process, and their function can be critical under abiotic stress [37]. CNMs can influence the synthesis or degradation of chlorophylls in a concentration-dependent manner, and make the photosynthesis process more efficient [38], in addition to influencing other vital biological processes such as proliferation cell, cytoskeletal redox processes, and stress responses associated with chloroplast development and protection [39]. The interaction of CNMs with plant cells can trigger oxidative stress responses through the generation of ROS and, consequently, activate the antioxidant defense system of plants (production of antioxidant compounds) to prevent the degradation of lipids, DNA, and proteins [26].

NMs have the potential to be used as elicitors or biostimulants for the induction of bioactive compounds in plants since they positively modify the expression of genes involved in the biosynthesis of secondary metabolites [13]. These metabolites are also capable of mitigating the limitations associated with saline stress [9]. At low concentrations, NMs activate the aquaporin channel and promote water and nutrient absorption [14] while at high doses, NMs promote the production of free radicals that induce oxidative stress and cell damage [13]. Therefore, it is of the utmost importance to define the appropriate doses to achieve the desired effect on the plants and to avoid possible negative impacts.

Some authors mention that the responses of plants exposed to abiotic stresses, combined with some biostimulant, are different from those observed when applied separately. The combination of elicitors can be synergistic or antagonistic and, consequently, it translates into a relief or a total failure of the plants to survive under biotic or abiotic stresses [20].

Several authors have reported the use of CNMs in seed priming and in the induction of tolerance to environmental stresses. Hatami et al. exposed *Hyoscyamus niger* L. seeds with single-walled CNTs (SWCNTs) (50–800 μg mL^−1^) under different levels of drought stress (0.5–1.5 MPa) [26]. The results showed that with low concentrations of SWCNTs, tolerance can be induced in seedlings against low levels of drought by improving water absorption and activating the plant defense system (SOD, POD, CAT, and APX) and the biosynthesis of proteins, phenolics, and specific metabolites such as proline. A reduction in oxidative damage indices, including H_2_O_2_, malondialdehyde content, and electrolyte leakage, was also observed. Abdel Latef et al. evaluated the priming of lupine plant seeds (*Lupinus termis* L.) with different concentrations of ZnO NPs (20, 40, and 60 mg L^−1^) and exposed to NaCl (150 mM) [40]. Salinity stress increased the content of organic solutes (soluble sugar, soluble protein, total free amino acids, and proline), total phenols, MDA, ascorbic acid, and Na and the activities of SOD, peroxidase (POD), and APX in stressed plants compared to control plants. Shafiq et al. investigated the effects of priming wheat seeds with fullerene (0, 10, 40, 80, and 120 mg L^−1^) under saline stress (150 mM NaCl) [4]. The results showed an increase in enzymatic activities, a reduction in the content of photosynthetic pigments (chlorophyll *a* and *b*, and carotenoids), and an alteration in ion absorption. 

Fan et al. reported that multiple-walled CNTs (MWCNTs) positively affected the photosynthesis system of plants by stimulating the electron transport rate and the photochemical quantum yield of photosystem II by up to 12% compared to the control [41]. CNTs improve the functioning of photosynthetic machinery because they can be integrated into the outer lipid envelope of chloroplasts; its semiconductor capacity triples photosynthetic activity by means of increased electron transport. Chloroplasts exposed to SWCNTs stimulate photosynthetic activity and increase electron transport flux by increasing photoabsorption by chlorophylls [42]. Baz et al. evaluated the priming of lettuce seeds (*Lactuca sativa* L.) with carbon nanoparticles (CNPs) that are soluble in water (0.3%) and under saline stress (150 mM NaCl). Pretreatment with CNPs promoted the accumulation of the total chlorophyll content of the seedlings grown under saline stress [43]. Joshi et al. applied MWCNTs (90 µg mL^−1^) in oats by means of the seed priming method and reported an increase in the chlorophyll content (57%) while the photosynthetic activity increased by 15%, thereby resulting in a better performance [44]. Joshi et al. used MWCNTs (70, 80, and 90 μg mL^−1^) to prime rice seeds. As a result, it was observed that the plants treated with MWCNTs had denser stomata and longer roots, resulting in faster growth and facilitating the absorption of water and minerals, thus increasing the crop yield [24]. In addition, the chlorophyll content and photosynthetic activity were improved. Gohari et al. evaluated COOH-functionalized MWCNTs (MWCNTs-COOH) (0, 25, 50, and 100 mg L^−1^) in sweet basil (*Ocimum basilicum* L.) seedlings under saline stress (50 and 100 mM NaCl) [45]. The results showed that the application of MWCNTs-COOH at an optimal concentration (50 mg L^−1^) ameliorated the negative effects of salinity stress by increasing the content of chlorophylls and carotenoids and inducing enzymatic antioxidant components, such as APX, CAT, and guaiacol peroxidase, and non-enzymatic-like phenols. Zhao et al. reported that 1000 μg L^−1^ of graphene oxide (GO) in *Arabidopsis thaliana* seedlings under saline stress (200 mM) increased the H_2_O_2_ content. Salinity induced higher generation of ROS (48.1% O_2_^•−^ and 62.2% H_2_O_2_) and lipid peroxidation (40.8% MDA) [46].

Park and Ahn reported a decrease in the total protein content in carrot seeds when evaluating the inclusion of CNTs (0–2000 mg L^−1^) for 5 days. Both authors suggested that crops with low protein levels could be more sensitive to certain types of stress, in addition to the fact that their expression or stability depends on the tissue or stage of development of the culture under study [47].

Fruit quality parameters for tomatoes include sugar content, pH, electrical conductivity, and titratable acidity, and reduced sugar content and firmness determine freshness and storage stability [17]. Saline stress (≥4 mS cm^−1^) affects the formation, development, maturation, and quality of tomato fruits [20]; causes osmotic stress and the active accumulation of solutes [48]; and disrupts ion homeostasis [49]. Variation in the levels of K^+^ modifies the levels of organic acids (citric and malic) in tomato fruits [50]. Meanwhile, the low levels of Ca^2+^ caused by the low translocation of Ca^2+^ towards the fruit negatively affect the firmness of the fruit and, consequently, its shelf life due to the low content of calcium pectates in its middle sheet [51]. 

The EC of fruits can be increased under saline stress due to a reduction in the size of the fruits, low accumulation of water, and a higher solute content [52]. The increase in the pH values in tomato fruits can be attributed to the imbalance between K^+^/Na^+^ and SO_4_^2−^/Cl^−^ ions, which maintain the stability of the pH of tomato fruits [53]. A decrease in ORP indicates a better quality of the fruit since it can translate into greater antioxidant potential [54]. Titratable acidity is a parameter that decreases in parallel with the evolution of fruit maturity since organic acids are used as a substrate in the respiration process. The seed priming with CNMs and the saline stress modified the quality parameters in tomato fruits. Morales-Díaz et al. suggested that when plants are subjected to abiotic stresses, it is possible for the presence of NPs and NMs to interact either synergistically or antagonistically, causing adverse responses in plants [55].

## 4. Materials and Methods

### 4.1. Plant Material and Carbon Nanomaterials

Tomato seeds “Pony Express F1” (Harris Moran, Davis, CA, USA), saladette type, and determinate growth, were used to develop the experiment. The tomato seeds were sterilized in a 2% solution of sodium hypochlorite for 5 min, and rinsed 5 times with distilled water [56].

Two carbon allotropes were used: carbon nanotubes (CNTs) and graphene (GP). The CNTs were multilayer (5 layers), approximately 95% pure, 30–50 nm in diameter, and 10–20 µm long (Nanostructured & Amorphous Materials, Inc., Katy, TX, USA). The graphene (GP) was multilayer (10–12 layers), with a purity of 97%, a diameter of 2 µm, and a thickness of 8–12 nm (Cheap Tubes Inc., Grafton, VT, USA).

### 4.2. Seed Treatment

The treatments consisted of three different concentrations of the two types of carbon nanomaterials based on the work of López-Vargas et al. [56]: 50, 250, and 500 mg L^−1^. In total, 40 tomato seeds were immersed in 20 mL of solution with the nanomaterial, and they were subsequently sonicated for 10 min in an ultrasonicator (sonicator Q500, QSONICA, Melville, NY, USA) as suggested by Ratnikova et al. [36]. Additionally, two controls that contained only distilled water were evaluated: one control comprised the non-sonicated seeds (SWS) and the other one the sonicated seeds (SS). All treatments were stored at room temperature for 24 h in their corresponding solutions to allow imbibition with the nanomaterials in the seeds. In contrast to López-Vargas et al., here, the seedlings employed were developed under saline stress conditions [56].

### 4.3. Crop Development

After 24 h of imbibition, the treated seeds were sown in polystyrene trays, where the seedlings developed for 30 days. After this time, the transplant was carried out in black polyethylene bags with a capacity of 14 L. A mixture of perlite–peat moss in a 1:1 ratio was used as substrate. 

To evaluate the impact of treatments, two experiments were established in the Antonio Narro Autonomous Agrarian University facilities (25°23′36″ N, 101°00′02″ W): one was developed without any stress condition and in the other, the plants were subjected to saline stress from transplantation and throughout the development of the crop (160 days from transplantation). To induce salinity stress, 50 mM NaCl was always added to the nutritive solution.

A directed irrigation system was used through which irrigation was applied, and Steiner nutritive solution was used to provide nutrition to the crop [57]. The pH was adjusted to 6.5 with sulfuric acid each time the nutritive solution was prepared. The electrical conductivity (EC) of the solutions was 1.9–2.5 mS cm^−1^ for the nutritive solution without sodium chloride (NaCl) and 5.5–7.5 mS cm^−1^ for the nutritive solution with NaCl.

### 4.4. Physico-Chemical Analysis of the Fruits

For these analyses, six fruits were selected for each treatment. They were washed with distilled water, and it was verified that they did not present damage, were of uniform size, and were in a state of maturity 5 (light red) according to the visual color scale of the USDA [58]. The fruits were collected 70 days after transplantation and from the second cluster, where each fruit was from a different plant. The hydrogen potential (pH), electrical conductivity (EC), total soluble solids (TSS), firmness, oxidation-reduction potential (ORP), and titratable acidity (TA) were measured as described in Lopez-Vargas et al. [59].

### 4.5. Antioxidant Compounds and Proteins

For this, samples of the leaves and fruits were collected at 65 days after transplantation, at around noon. The collected leaves were fully developed young leaves, and the fruits presented the same characteristics as those used for the physico-chemical analysis. The samples were collected on ice and stored at a temperature of −20 °C. Later, they were lyophilized and macerated until a fine powder was obtained.

For the biochemical analyses, two different extractions were carried out: one to determine the hydrophilic compounds, as described by Abdel Latef and Tran [60], and another to determine the lipophilic compounds, according to Nagata and Yamashita [61].

The content of chlorophylls and carotenoids was determined according to Nagata and Yamashita [61]. Vitamin C was determined according to Hung and Yen [62]. Reduced glutathione (GSH) was determined by reaction with 5,5 dithio-bis-2 nitro benzoic acid (DTNB) according to Xue et al. [63]. The results are expressed in mmol GSH EQ 100 g^−1^ DW. The content of total phenols was determined according to Yu and Dahlgren [64]. Total phenols are expressed in mg EQ of gallic acid per gram of DW. The flavonoid content was determined according to Arvouet-Grand et al. [65]. The results are expressed in mg EQ of quercetin per gram of DW.

The antioxidant capacity was determined using the DPPH (1,1-diphenyl-2-pricrilhydrazil) method for both hydrophilic and lipophilic compounds. The total antioxidant capacity (TAC) was obtained by adding the hydrophilic and lipophilic compounds. The results are expressed as mg EQ of ascorbic acid per gram of DW [66]. The quantification of proteins was determined using the technique of Bradford [67] and the results are expressed in mg g^−1^ of DW.

### 4.6. Stress Biomarkers

The determination of hydrogen peroxide (H_2_O_2_) was performed according to the methodology described by Velikova et al. [68], and the results are expressed as μmol g^−1^ of DW. Malondialdehyde (MDA) was determined according to Velikova et al. [68] and the results are expressed as nmol g^−1^ of DW. The determination of ion leakage was carried out according to Jiang and Zhang [69] and the results are presented as % ion loss.

### 4.7. Enzymatic Activity

Ascorbate peroxidase (APX) activity (EC 1.11.1.11) was assessed according to Nakano and Asada [70]. The glutathione peroxidase (GPX) activity (EC1.11.1.9) was determined according to Xue et al. [63]. The catalase activity (CAT) (EC 1.11.1.6) was determined according to Dhindsa et al. [71]. Superoxide dismutase (SOD) activity (EC. 1.15.1.1) was determined using the SOD Cayman 706002^®^ kit. The activity of phenylalanine ammonia lyase (PAL) (EC 4.3.1.5) was determined according to Sykłowska-Baranek et al. [72].

### 4.8. Statistical Analysis

The experiment was set up using a completely randomized design and six replicates per treatment were analyzed. The homogeneity of the data was analyzed using the Shapiro–Wilks method. The one-way analysis of variance and the Fisher LSD mean comparison test (α = 0.05) were performed in the Infostat software (v2020) (Universidad Nacional de Córdoba, Córdoba, Argentina) according to López-Vargas et al. [50].

## 5. Conclusions

The impact of seed priming with CNMs on the biochemical responses and content of bioactive compounds in tomato plants grown under salinity stress conditions was evaluated. The results showed that CNMs can function as elicitors and activate the antioxidant defense system of tomato plants.

The use of CNMs in the nano-priming of tomato seeds had a positive impact on the accumulation of proteins and antioxidant compounds in the leaves. However, this effect was more evident under NaCl stress since the combination of CNMs + NaCl potentiated the increase in enzymatic and non-enzymatic antioxidant compounds.

The increase in antioxidant compounds seems to be consistent since the use of CNMs improved the antioxidant capacity of the fruits by increasing lycopene and glutathione. While the combination of CNMs and salinity stress increased lycopene, ascorbic acid, phenols, glutathione, and flavonoids.

CNMs can be used as eliciting agents in seed priming to improve the antioxidant system of plants and the quality of tomato fruits. According to the results, CNTs at concentrations of 250–500 induced the highest content of bioactive compounds. However, it is necessary to perform more studies to define the optimal treatments in terms of the CNMs used and their concentration to obtain the desired positive effects while also considering the impacts on growth and fruit yield. Furthermore, CNMs in nano-priming are not applied directly to the soil, so the dispersion of large amounts of NMs in ecosystems can be avoided.

This technology could be very useful in agriculture since it can be easily applied, in addition to the fact that the amount of CNMs necessary for seed priming is small, so it is relatively inexpensive.

## Data Availability

Not applicable.
